# Copepod Prey Selection and Grazing Efficiency Mediated by Chemical and Morphological Defensive Traits of Cyanobacteria

**DOI:** 10.3390/toxins12070465

**Published:** 2020-07-21

**Authors:** Luciana M. Rangel, Lúcia H. S. Silva, Elisabeth J. Faassen, Miquel Lürling, Kemal Ali Ger

**Affiliations:** 1 Laboratório de Ficologia, Museu Nacional, Departamento de Botânica, Universidade Federal do Rio de Janeiro, 20940-040 Rio de Janeiro, Brazil; luciana.rangel@gmail.com (L.M.R.); luciahssilva@gmail.com (L.H.S.S.); 2 Wageningen Food Safety Research, Wageningen Research, Akkermaalsbos 2, 6708 WB Wageningen, The Netherlands; els.faassen@wur.nl; 3 Department of Environmental Sciences, Aquatic Ecology and Water Quality Management Group, Wageningen University, Droevendaalsesteeg 3a, 6708 PB Wageningen, The Netherlands; 4 Center for Coastal Limnological and Marine Studies (CECLIMAR), Campus Litoral Norte, Universidade Federal de Rio Grande de Sul, 95625-000 Imbé, Brazil

**Keywords:** cyanotoxin, harmful algal bloom, neurotoxin, predator defense, functional trait

## Abstract

Phytoplankton anti-grazer traits control zooplankton grazing and are associated with harmful blooms. Yet, how morphological versus chemical phytoplankton defenses regulate zooplankton grazing is poorly understood. We compared zooplankton grazing and prey selection by contrasting morphological (filament length: short vs. long) and chemical (saxitoxin: STX- vs. STX+) traits of a bloom-forming cyanobacterium (*Raphidiopsis*) offered at different concentrations in mixed diets with an edible phytoplankton to a copepod grazer. The copepod selectively grazed on the edible prey (avoidance of cyanobacteria) even when the cyanobacterium was dominant. Avoidance of the cyanobacterium was weakest for the “short STX-” filaments and strongest for the other three strains. Hence, filament size had an effect on cyanobacterial avoidance only in the STX- treatments, while toxin production significantly increased cyanobacterial avoidance regardless of filament size. Moreover, cyanobacterial dominance reduced grazing on the edible prey by almost 50%. Results emphasize that the dominance of filamentous cyanobacteria such as *Raphidiopsis* can interfere with copepod grazing in a trait specific manner. For cyanobacteria, toxin production may be more effective than filament size as an anti-grazer defense against selectively grazing zooplankton such as copepods. Our results highlight how multiple phytoplankton defensive traits interact to regulate the producer-consumer link in plankton ecosystems.

## 1. Introduction

Anti-grazer defenses of phytoplankton regulate the structure and function of aquatic ecosystems and are associated with the accumulation of toxic or otherwise harmful blooms [1,2,3]. Bloom-forming cyanobacteria are abundant in estuarine and freshwater ecosystems, and cause economic, environmental, and public health problems globally [4,5]. Harmful cyanobacterial blooms are predicted to increase in occurrence, duration, and magnitude due to anthropogenic eutrophication and global climate change [6,7]. Ecologically, such blooms uncouple the link between primary producers and zooplankton consumers, and therefore inhibit the transfer of carbon and energy to higher trophic levels [8,9].

Reduced zooplankton grazing on bloom-forming cyanobacteria has been linked to putative anti-grazer defenses such as morphological (e.g., filament or colony formation) or chemical (e.g., toxin production) traits [10,11]. High-density blooms usually consist of large-sized filaments or colonies, which are morphological traits that can act as mechanical grazing deterrents [12,13]. In addition to morphologic defenses, several species and strains produce toxic compounds [14,15], including neurotoxins, which may also act as chemical anti-grazer defenses [16,17]. Many bloom-forming cyanobacteria species show high phenotypic plasticity in these defensive traits [18,19,20]. Yet, most work on cyanobacteria grazing is done with cultured unicellular *Microcystis* as the model species, which limits knowledge on the effects of morphological defenses [12,21]. Little is known, therefore, about the effect of multiple trait variability (e.g., chemical and morphological) on grazing losses, which may be a key regulator of cyanobacterial blooms.

Grazing selectivity is a key functional trait that mediates the response of zooplankton to phytoplankton defenses [22,23]. Active selection occurs when a grazer can choose among different prey items and when grazing on a given prey is different than what would be expected based on its relative abundance [24]. In contrast, generalist grazers non-selectively ingest all prey in within the edible size range at rates proportional to the relative abundance of each prey [25].

Cyanobacterial blooms in freshwaters typically lead to the exclusion of large generalist grazers, which are often replaced by smaller and selectively grazing copepods [21,26,27]. Both toxin production and filamentous morphology have negative effects on large and generalist grazers such as *Daphnia* either via toxin exposure or feeding inhibition [12,13,28,29]. In contrast, selectively feeding copepods are better adapted to avoid lower quality prey and can overcome toxin exposure by grazing on edible prey [30,31,32]. Moreover, positive selection by copepods for edible phytoplankton may promote blooms of inedible or toxic phytoplankton [33,34] including cyanobacteria [35]. Thus, understanding the role of cyanobacterial defensive traits on copepod grazing and prey selection is necessary to predict the ecology of blooms.

The bloom-forming cyanobacterium *Raphidiopsis raciborskii* (formerly *Cylindrospermopsis*) is increasingly reported in freshwater systems globally [36,37,38,39]. This species is characterized by solitary free-floating filaments [40,41], with significant filament length variability of ~2 orders of magnitude in natural populations and isolated strains maintained in laboratory conditions [18,42,43]. The toxicity of *Raphidiopsis* blooms is also variable and some strains can produce neurotoxins such as saxitoxins (STX) or cyanotoxins such as cylindrospermopsin (CYN) [37,43,44,45]. Considering high *Raphidiopsis* genotype or phenotype variability in nature, how morphological versus chemical defenses affects prey selection in copepods—the dominant grazer during blooms of this cyanobacterium—remains unknown [27,46,47,48,49].

A major obstacle in understanding the role of phytoplankton defensive traits arises because most experiments consider single trait variation even though several defensive traits may exist for a given species. For example, copepods are known to graze on *Raphidiopsis*, and chemical traits (toxins such as STX and CYN) have been linked to lower grazing rates [48,50]. Yet, the role of filament size was not established in these experiments. In a recent experiment, we compared the relative role of chemical (i.e., absence or presence of STX) versus morphological (i.e., different filament lengths) defenses on copepod grazing using pure diets of *Raphidiopsis* [51]. This experiment showed that both filament size and STX content reduced grazing pressure though the latter was more important. This was, however, a preliminary step for testing both defensive traits, and the observation was based on assays with single-prey diets that hardly represent natural conditions where edible prey is available [21,52].

Accordingly, here we build on the work of Rangel et al. [51] in a parallel study by testing the effect of *Raphidiopsis* grazer defenses on copepod prey selection in the presence of edible prey. Specifically, we compared the effect of morphological (filament size: long vs. short) and chemical (saxitoxin producing vs. non-producing) defenses of *Raphidiopsis* on copepod grazing and prey selection across a gradient of the cyanobacterium offered in mixed diets with edible prey to a common Eurasian copepod (*Eudiaptomus gracilis*). We asked how each defensive trait would change copepod grazing and hypothesized that the copepod (1) would positively select the edible prey over the cyanobacteria; (2) have stronger avoidance of the toxic or longer cyanobacteria filaments; and that 3) increased cyanobacterial dominance would not interfere with the grazing of edible prey.

## 2. Results

### 2.1. Functional Response

**Figure 1:**
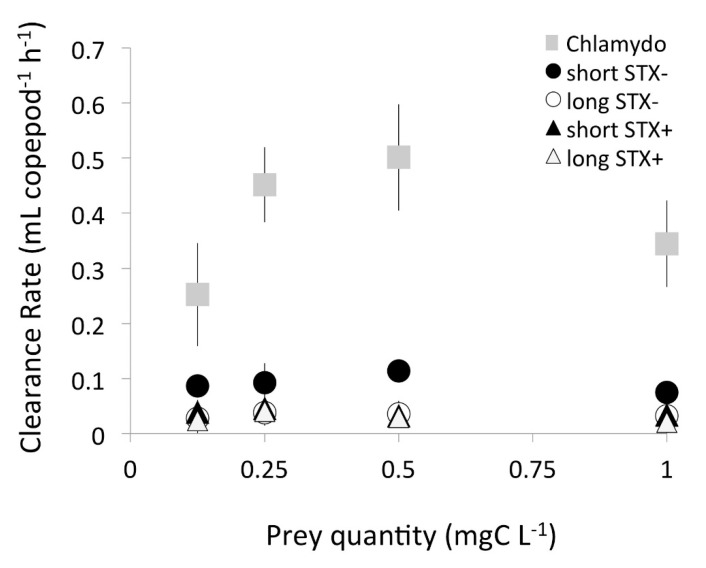
The functional response of the copepod *Eudiaptomus* showing mean clearance rate for the edible prey (Chlamydo: *Chlamydomonas*); and the four different strains and morphotypes of the cyanobacterium *Raphidiopsis* offered as the sole food source across different concentrations. Short or long refer to *Raphidiopsis* filament size, while the STX refers to strains that produce (STX+) or do not produce saxitoxin (STX-). Error bars show standard deviation (*n* = 4).

The copepod grazed on all prey in the single diet experiments, but at different rates (Figure 1). Clearance rates (CR) were different among prey types and grazing on *Chlamydomonas* was significantly higher (by a factor of 3–10×) compared to all the other types of the cyanobacteria across all prey concentrations (Table 1A). Among the *Raphidiopsis* diets, CR_R_ was significantly higher for the short STX- strain, while grazing on all three other strains were similar (Table 1B). Mean CR on the edible prey *Chlamydomonas* (CR_C_) followed a type-3 functional response as it increased with higher prey concentration and peaked at 0.5 mg C L^−1^. In contrast, mean CR on *Raphidiopsis* (CR_R_) was low throughout different prey concentrations (Figure 1).

### 2.2. Grazing on Mixed Diets

**Figure 2:**
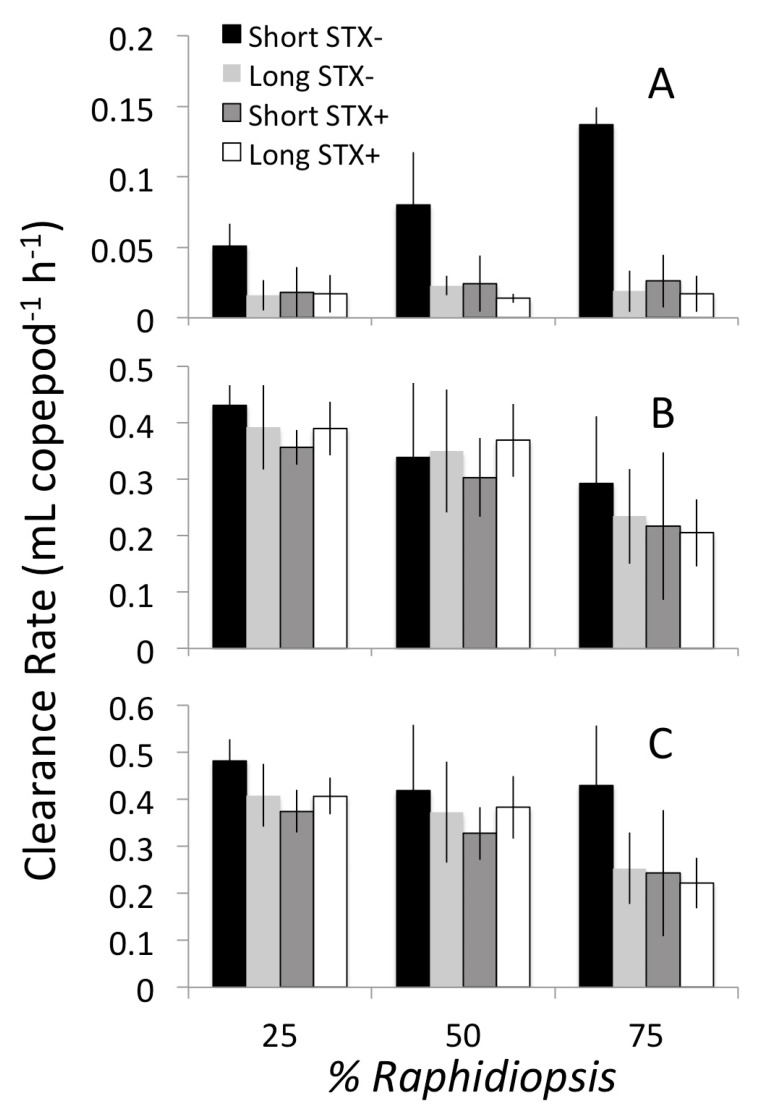
Mean clearance rate of the copepod *Eudiaptomus* grazing on mixed diets containing the edible prey (*Chlamydomonas*) and one of the *Raphidiopsis* strains across different biomass proportions of the cyanobacterium. Rates are shown for (**A**) *Raphidiopsis*; (**B**) *Chlamydomonas*; and (**C**) the total on both phytoplankton prey in treatments receiving Raphidiopsis with contrasting defensive traits (filament size: short or long; saxitoxin production: STX+ or STX-). Error bars show standard deviation (*n* = 4).

The copepod grazed between 2 and 10× more *Chlamydomonas* than *Raphidiosis* (Figure 2) and CR_R_ was significantly less than CR_C_ across all treatments (*p* < 0.001). The effect of filament size (short vs. long), toxin production (STX+ vs. STX-), and dietary proportion of *Raphidiopsis* (%R) on prey specific grazing rates is shown in Table 2. Grazing on the cyanobacterium (CR_R_) was significantly reduced by a factor of 3–6× with the STX+ or longer filaments compared to the short STX- treatment (Table 2A). Furthermore, increased dietary proportion of *Raphidiopsis* had a significant positive effect on how much this prey was grazed (i.e., CR_R_).

These effects of filament size, toxin production, and dietary proportion of *Raphidiopsis* on CR_R_, however, were not observed when the short STX- strain was removed from the analysis. Indeed, the copepod grazed similarly, albeit at very reduced rates (Figure 2A), on the other three *Raphidiopsis* treatments without any effect of filament size (*p* = 0.226), toxin production (*p* = 0.551), or %R (*p* = 0.517). Hence, the effect of filament size on CR_R_ was limited to the strains that did not produce saxitoxin (STX-). Taken together, toxin production and longer filament size both reduced grazing on the cyanobacterium but the effect of filament size was secondary to that of toxin production (i.e., only observed when comparing among the STX- strains).

Grazing on *Chlamydomonas* (CR_C_) was similar in diets with contrasting cyanobacterial defensive traits (Figure 2B), and neither filament size nor STX production had a significant effect on the CR_C_ (Table 2B). Yet, increasing dietary proportion of *Raphidiopsis* significantly reduced CR_C_ (Table 2B). Consequently, the overall total clearance rate (i.e., CR_Total_ = CR_R_ + CR_C_) was also inversely proportional to the relative dominance of the cyanobacterium, which had a significant negative effect on overall grazing rates (Figure 2C, Table 2C). The short STX- treatment, however, was an exception. When analyzed without the other treatments, total clearance rate in the short STX- treatment was only marginally affected by the relative dominance of *Raphidiopsis* (*p* = 0.077). In contrast, for the long STX-, short STX+, and long STX+ treatments, the relative dominance of *Raphidiopsis* had a significant negative effect on CR_Total_. Thus, the effect of the relative dominance of the cyanobacterium on CR_Total_ was strain-specific (Table 2D). Moreover, although CR_Total_ declined significantly in treatments with the STX+ strains (Figure 2C, Table 2C), this effect was not observed when the short STX- treatment data was removed from the analysis (Table 2D). Overall, both defensive traits (filament size and toxin production) significantly affected copepod grazing on the cyanobacterium (CR_R_), but not the ingestion of the edible prey (i.e., CR_C_). The latter, however, was negatively affected by cyanobacterial dominance.

### 2.3. Copepod Prey Selection: The Selectivity Coefficient for Raphidiopsis (α_R_)

**Figure 3:**
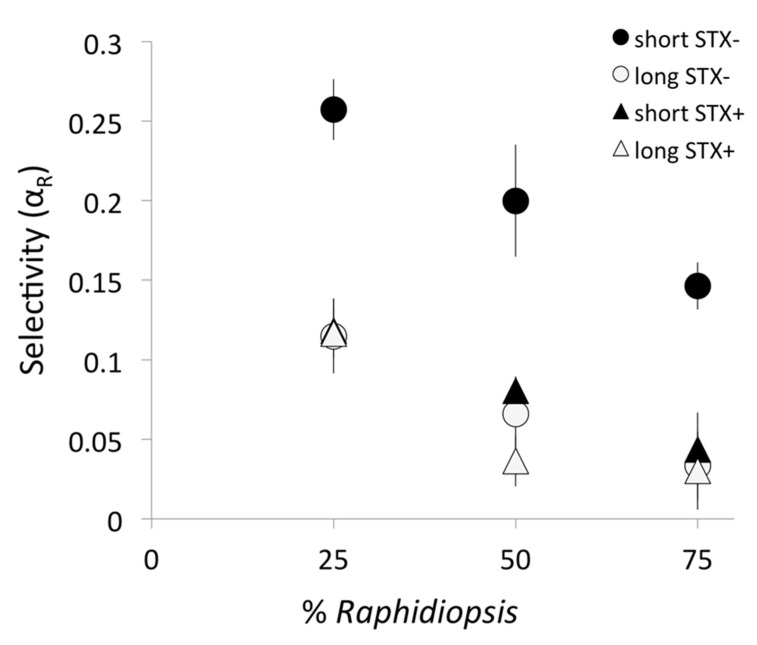
Mean selectivity coefficient of the copepod *Eudiaptomus* for the cyanobacterium *Raphidiopsis* (αR) on mixed diets containing the edible prey (*Chlamydomonas*) and one of the *Raphidiopsis* strains across different biomass proportions of the cyanobacterium with contrasting defensive traits (filament size: short or long; saxitoxin production: STX+ or STX-). Error bars show standard deviation (*n* = 4). Values below 0.5 indicate active avoidance of the cyanobacterium; values closer to zero indicate stronger avoidance (see text for details).

In all treatments, the copepod positively selected the edible prey *Chlamydomonas*, and actively avoided *Raphidiopsis* (Figure 3, α_R_ < 0.5, see methods for details). Yet, copepods in the short STX- treatments showed the weakest avoidance of the cyanobacterium as indicated by the significantly higher α_R_ between 0.15 and0.27 (Figure 3, Table 3A). In contrast, copepods avoided the other three strains of the cyanobacterium more strongly as shown by the significantly lower α_R_ values below 0.12. Hence, selective avoidance was weakest for the short STX- filaments and strongest for the other three cyanobacterial strains. Notably, the effects of the defensive traits (STX or filament size) on grazing selectivity was not observed when the short STX- strain was removed from the analysis (Table 3B). Hence, the copepod strongly avoided the long STX-, short STX+, and long STX+ strains but did so at similar efficiency. Filament size, therefore, had an effect on cyanobacterial avoidance only in the absence of toxin (i.e., STX- treatments). Toxin content, however, significantly increased cyanobacterial avoidance (i.e., reduced α_R_) in both short and long filament size treatments (Table 3). Finally, increasing cyanobacterial dominance reduced the selectivity coefficient regardless of their defensive traits (Figure 3, Table 3).

## 3. Discussion

We compared the role of phytoplankton defensive traits (i.e., morphological vs. chemical) on copepod grazing behavior and prey selection using strains of the bloom-forming cyanobacteria *Raphidiopsis* with contrasting morphology (short vs. long filaments) and toxin production (STX+ vs. STX-) as a model species offered together with edible prey (*Chlamydomonas*). As expected, the copepod selected against all strains of the cyanobacterium and grazed selectively on the edible prey. A major result was the trait-specific avoidance of the cyanobacterium: longer filament size increased avoidance only in the absence of toxin production while toxin production increased avoidance regardless of filament size. Moreover, and contrary to expected, grazing on edible food was inversely proportional to *Raphiodipsis* dominance. Another key result, therefore, was that the dominance of filamentous cyanobacteria such as *Raphidiopsis* may inhibit copepod grazing despite selective grazing of edible prey. Overall, results emphasize the trait-specific manner in which copepod prey selection responds to filament size and toxin production. The implications of these results for copepod grazing behavior and the role of defensive traits in harmful algal bloom dynamics are discussed below.

Copepod selective feeding is regulated by prey encounter rate and subsequently the remote detection and individual handling of prey items via chemosensory cues [53]. Encounter rates depend on the abundance, size, and shape of planktonic prey [54]. Once prey is encountered, copepods use chemosensory cues to individually identify and ingest the highest quality (i.e., edible and nutritious) items available while actively avoiding low quality (i.e., inedible, nutritionally poor, or toxic) items [32,55]. That the copepod *Eudiaptomus* avoided all strains of *Raphidiopsis*, albeit to varying degrees, supports previous studies showing that this cyanobacterium is a low-quality prey for copepods [50,56]. Cyanobacteria are known to be low-quality prey not only due to their defensive traits but also because they lack several essential nutrients [57,58]. The trait-specific avoidance of *Raphidiopsis* we observed, however, indicate that both toxin production and filament size are important cues for copepods.

We found that STX production increased copepod avoidance of short or long *Raphidiopsis* filaments while longer filament size increased copepod avoidance only for the STX- strain. Taken together, these results support findings of the previous Rangel et al. [51] study and suggest that i) saxitoxin production was the primary defensive trait because it was independent of filament size and ii) longer filaments were a secondary defensive trait operating in the absence of saxitoxin production. Indeed, saxitoxin is considered a chemical phytoplankton defense, with clear negative effects on zooplankton fitness and grazing [59,60]. Yet, almost all copepod studies with saxitoxin producing phytoplankton come from marine environments with dinoflagellate prey [2,55]. In a rare study with cyanobacteria, saxitoxin production by *Raphidiopsis* has also been associated to selective avoidance by the calanoid copepod *Boeckella* [50]. Hence, our results support that saxitoxin can be a defensive trait against copepod grazing for cyanobacteria in addition to dinoflagellates.

That larger filament size also increased *Raphidiopsis* avoidance, albeit in the absence of saxitoxin production, is evidence for the defensive role of this trait. Filament size is expected to be a defensive trait when it provides a grazer refuge by growing larger than the edible prey size for a given zooplankton [61]. Filaments larger than the edible size can clog the filtering apparatus of cladocerans [13,28] or increase prey handling time for copepods [61,62,63]. Yet, within the edible prey size range, filament or chain formation increases the encounter rate compared to unicellular phytoplankton [53,64,65]. Thus, copepods are expected to ingest filaments within the edible size range more than unicellular phytoplankton, especially if it is a good quality prey [53,64]. The optimum prey size for diaptomid calanoid copepods including *Eudiaptomus* is between 10 and 50 μm [66]. Hence, the shorter STX- *Raphidiopsis* strain (31 ± 20 μm) was within the edible prey size range while the longer STX- strain (130 ± 33 μm) was outside the optimal prey size. That the former was less avoided than the latter is likely explained by increased handling time for longer filaments.

In addition to the defensive traits, *Raphidiopsis* dominance was a key driver of prey specific and total grazing rates. In the current design, grazing on edible non-toxic prey is expected to be proportional to its relative abundance [54,67]. In contrast, grazing on non-edible or toxic prey is unrelated to its relative abundance as the grazer is actively avoiding ingestion of this prey [24]. The current results, therefore, confirm that the STX+ or longer filaments of *Raphidiopsis* were poor quality and relatively inedible. These strains were grazed upon at similarly low rates in the previous Rangel et al. study [51] with pure *Raphidiopsis* diets, and taken together, our results show that avoidance of these strains was independent of the availability of edible prey. This is often the case with copepod grazing on unicellular toxic cyanobacteria such as *Microcystis* [31,67]. In contrast, grazing on the short STX- strain was positively proportional to its abundance, indicating that this strain was more edible than the other three strains of *Raphidiopsis*. When *Raphidiopsis* was dominant (i.e., 75% of total food), clearance rate on the short STX- strain was relatively high (0.14 mL copepod^−1^ h^−1^), which was ~ 50% of the rate for edible prey. Yet, with increasing availability of edible prey, clearance rate on the short STX- strain declined to 0.05 mL copepod^−1^ h^−1^. In contrast, regardless of edible prey availability, grazing on the remaining three strains was always low (<0.03 mL copepod^−1^ h^−1^), which was <10% of the rate for edible prey. Thus, non-toxic filaments of cyanobacteria within the edible prey size may be readily ingested during blooms.

Grazing on the edible prey (*Chlamydomonas*), and consequently total grazing, were both reduced by increasing dominance of *Raphidiopsis*. Hence, contrary to our hypothesis, *Raphidiopsis* dominance reduced the efficiency of grazing on edible prey despite selective grazing. This contrasts with previous observations from experiments with the toxic cyanobacteria *Microcystis* [31,67] and *Nodularia* [68], whose abundance did not reduce copepod grazing on alternative edible prey. Although increasing prey encounter rate, filamentous phytoplankton can also interfere with copepod grazing due to increased handling time compared to unicellular prey [64]. Several studies indicate that copepods cut longer cyanobacterial filaments into smaller pieces during grazing [27]. Such “shredding” may increase the handling time of individual filaments and consequently reduce the encounter rate with edible prey during blooms of harmful phytoplankton [3,53]. Moreover, that the short STX+ strain reduced total grazing similar to the longer (STX+ or STX-) filaments is evidence that toxin production may also interfere with copepod grazing at higher *Raphidiopsis* dominance. Thus, as with some cladocerans, cyanobacterial toxin production may also inhibit copepod grazing efficiency when edible prey is scarce [69]. Notably, increased *Raphidiopsis* dominance did not significantly reduce total grazing in the short STX- treatment, which was compensated by increased grazing on the short STX- strain itself. This is evidence that the short STX- strain did not interfere with copepod grazing efficiency. Overall, our results emphasize that in contrast to *Microcystis* and *Nodularia*, the dominance of *Raphidiopsis*, especially toxin-producing or longer filaments, may reduce copepod grazing efficiency in nature.

The observed negative relation between *Raphidiopsis* dominance and the selectivity coefficient for this cyanobacterium (αR) is likely an artifact of our method to measure clearance rates. The assumption to accurately measure selectivity is an unchanging proportion among prey during the grazing period [70]. Due to active avoidance of *Raphidiopsis*, however, the proportion of this cyanobacterium increased during the experiment. Consequently, in our setup, the αR values are likely overestimated, especially for the 25% *Raphidiopsis* treatment [71]. Nevertheless, the effect of toxin production or filament size on αR is independent of any overestimation of αR because differences among these treatments are conserved for a given proportion of *Raphidiopsis*. Hence, while αR may not be the perfect metric for selectivity in grazing experiments based on prey loss, conclusions about the effect of toxin production and filament size on selectivity are warranted in this setup [67].

A major challenge when testing the role of putative phytoplankton defenses is the difficulty of controlling for a single trait among different phytoplankton strains [1]. Indeed, although grazing on the short and long STX+ strains was similar, longer filaments also produced less saxitoxin. Thus, similar grazing on longer (less toxin) vs. shorter (more toxin) filaments may have been, at least partially, due to a potential trade-off between filament size and toxicity. The previous Rangel et al. study [51] with pure *Raphidiopsis* diets, however, showed that the presence–not concentration–of STX was likely the main factor that deterred grazing, and consequently, that filament size was secondary to toxin production as a grazer defense. Hence, while our results suggest a primary defensive role for toxin production, future work is necessary to resolve potential trade-offs among chemical versus morphological defensive traits. This might be possible by using mechanically shortened filaments with identical chemical traits including toxin production and nutrition. Our results could also be due, at least partially, to other unaccounted differences in the chemical traits of the *Raphidiopsis* strains including unidentified toxins [72,73,74] or nutrition [57,58]. Moreover, confirming the results obtained here with a larger pool of STX+ and STX- strains would corroborate the role of STX as an anti-grazer defense for *Raphidiopsis*.

That specific defensive traits of cyanobacteria act as anti-grazer cues for selectively grazing zooplankton has important implications for the producer-consumer link in aquatic ecosystems. In addition to being a key consumer in pelagic ecosystems, copepods are often the major zooplankton in ecosystems with harmful blooms of phytoplankton including cyanobacteria [21,47,75,76]. There is theoretical [33,34,77] and empirical [23,35] evidence that copepod selective grazing may promote blooms of harmful phytoplankton. Our results build on these predictions and suggest that cue-based selective copepod grazing may promote the dominance of cyanobacteria with longer filament size or toxin production. The defensive role of filament size we observed, however, may weaken with time due to copepod shredding of cyanobacterial filaments down to the edible size range [48]. Moreover, copepods can locally adapt to evolve tolerance to phytoplankton toxins [78]; are known to assimilate cyanobacterial carbon in nature [76,79,80,81]; and can be adapted to ingesting and detoxifying toxic cyanobacteria [31]. Thus, toxin-producing strains of *Raphidiopsis* may be ingested at higher rates depending on local adaptation. Despite toxin tolerance, however, copepods can still be expected to avoid bloom-forming cyanobacteria, especially when alternative nutritious prey is available, due to stoichiometric constraints [24,25].

## 4. Conclusions

Taken together, our results build on the preliminary Rangel et al. [51] study by showing that (i) toxin production and filament size are important regulators of copepod prey selection, and moreover, (ii) filamentous cyanobacteria can interfere with copepod grazing in a trait specific manner depending on the availability of alternative prey. Results emphasize that trait variation can mediate grazing pressure on filamentous cyanobacteria, resulting in 10-fold differences among clearance rates. Hence, shorter and non-toxin producing filaments of cyanobacteria, although still avoided, may be ingested at relatively high rates, especially during blooms. Yet, defensive traits of cyanobacteria may interfere with copepod grazing and therefore reduce copepod fitness due to limited energy intake during blooms when edible prey is scarce. This preliminary evidence suggests that neurotoxin production may be a more effective defense compared to filament size. Future efforts that simultaneously compare the effectiveness of multiple defensive traits can provide a more systematic understanding of trait-based phytoplankton defenses and subsequent effects on the producer-consumer link in pelagic ecosystems.

## 5. Materials and Methods

### 5.1. Phytoplankton Cultivation

The methods for phytoplankton and zooplankton cultures are described in detail in the previously published sister study Rangel et al. [51]. Briefly, two strains of the cyanobacterium *Raphidiopsis raciborskii* (i.e., *Raphidiopsis*) were obtained from the Laboratory of Ecophysiology and Toxicology of Cyanobacteria (LETC, Federal University of Rio de Janeiro, Brazil) and were used in this study. The saxitoxin producer LETC CYRF-01 (i.e., STX+) and LETC CS1, which had no saxitoxin or cylindrospermopsin production detected (i.e., STX-). The chlorophycean *Chlamydomonas reinhardtii* (NIVA-CHL13) and the cryptophycean *Cryptomonas pyrenoidifera* (NIVA 2/81) were obtained from the Norwegian Institute for Water Research (NIVA, Oslo, Norway). Stock cultures, with the exception of *Cryptomonas*, were maintained as semi-continuous batch cultures in modified WC medium in 300 mL Erlenmeyer flasks. Flasks were placed at 25 °C, under continuous orbital shaking (60 rpm), in a photoperiod of 14 h with a maximum intensity of 50 μmol photons m^−2^ s^−1^ of light. *Cryptomonas* was maintained in a chemostat under identical conditions as the other phytoplankton. Under these conditions, *Chlamydomonas* cells were spherical with a mean diameter of ≈10 µm. The cyanobacteria and chlorophyte (as edible prey) were used in grazing experiments. *Cryptomonas* was used to acclimate copepods and not in the grazing experiments because of the overlapping fluorescence signal with the cyanobacteria used to detect prey abundance [51].

### 5.2. Establishment of Short and Long Filament Cyanobacterial Morphotypes

As previously described [51], we grew the STX+ and STX- strains at 17 °C (for obtaining longer filaments) or 32 °C (for obtaining shorter filaments) for two months (all other conditions identical to stock cultures above). For each strain and morphotype, we calculated the mean filament length (± SD) based on 50 filaments. This resulted in two comparable long (STX+: 158 ± 66 µm; STX-: 130 ± 33 μm) filaments and two comparably short (STX+: 31 ± 20 µm; STX-: 55 ± 25 μm) filaments for each strain. Hence, there was a short- and long-filament morphotype for both the STX+ and STX- strain of cyanobacteria that were used in grazing assays to test the role of toxin production vs. filament size on copepod grazing.

### 5.3. Toxin Analyses of the STX+ and STX- Strains

Toxin production (saxitoxins: saxitoxin -STX, neosaxitoxin -NEO, decarbamoylsaxitoxin - dcSTX and decarbamoylneosaxitoxin - dcNEO; and gonyautoxins: GTX1-4, decarbomoyl gonyautoxin -dcGTX2-3; certified standards National Research Council, Canada) of the *Raphidiopsis* strains and morphotypes was performed by liquid chromatography-tandem mass spectrometry (LC-MS/MS; Agilent Technologies, Santa Clara, CA, USA), as previously described [51]. The short- or long- STX- strains did not produce any detectable toxin while both STX+ morphotypes produced a mixture of STX, dc STX, NEO, and dcNEO. The short and long STX+ strain produced a total of 1.09 × 10^−10^ and 1.21 × 10^−11^ µg mm^−3^ toxin per biovolume, respectively [51]. This resulted in a short and long STX+ strain to be used in the grazing assay.

### 5.4. Copepods Used for Grazing Experiments

Adults of the calanoid copepod *Eudiaptomus gracilis* were sampled from Lake Rauwbraken (The Netherlands) with a plankton net and returned to the laboratory, where they were subsequently acclimated to laboratory conditions for five days in Dutch Standard Water medium before the grazing experiment as described previously [51]. Copepods were fed *Cryptomonas* at a rate of 0.5 mg C L^−1^d^−1^ during this time. Only healthy (i.e., active and free of external parasites) adult copepods were used in the grazing experiments.

### 5.5. Copepod Functional Response to Different Prey

In order to define the prey concentration that maximizes grazing rates of the copepod, we designed grazing assays comparing the functional response of copepod clearance rate on the five different phytoplankton prey across a food concentration of 0.125, 0.250, 0.5 and 1 mg C L^−1^. The five different prey were *Chlamydomonas* (C) and the four *Raphidiopsis* strains described above (i.e., short STX-, long STX-, short STX+, long STX+). The functional response shows how grazing rates respond to the availability of a single prey and is useful for determining the total prey concentration to be used in the mixed-prey experiment (i.e., the concentration corresponding to the highest grazing rate) [53,67].

### 5.6. Copepod Selective Grazing in Mixed Prey Diets

In order to estimate the effect of cyanobacterial defenses (i.e., filament size and toxin production) on copepod prey selection, we designed grazing assays with mixed prey. For this, each strain of the cyanobacteria (i.e., short STX-, long STX-, short STX+, long STX+) was paired with the edible *Chlamydomonas*, resulting in four different diets (prey pairs). Moreover, to quantify potential effects due to changes in the relative dominance of the cyanobacterium (e.g., grazing inhibition) we also provided each prey pair at three different proportions corresponding to:

- 25% *Raphidiopsis* + 75% *Chlamydomonas*;
- 50% *Raphidiopsis* + 50% *Chlamydomonas*;
- 75% *Raphidiopsis* + 25% *Chlamydomonas*.

Thus, there were four different prey pairs crossed with three different proportions. Given that the highest mean grazing rate was observed with 0.5 mg C L^−1^ of edible prey in the functional response (see results), we used this as the total prey concentration for these mixed-prey experiments. By comparing grazing responses of each prey to its availability, this setup distinguishes differences in the quality or edibility among prey [10].

All grazing experiments were performed as explained previously [30]. Briefly, we calculated prey specific clearance rates (mL copepod^−1^ h^−1^) by comparing changes in prey specific chlorophyll-a concentrations in no-grazer controls vs. treatments with copepods over a two-hour grazing period (Supplementary Material, Figure S1). Prey specific chlorophyll-a concentration was measured by a PHYTO-PAM (HeinzWalz GmbH, Effeltrich, Germany) using a calibrated “blue” signal for the cyanobacteria and “green” signal for the *Chlamydomonas*. The grazing experiment took place in 24-well plates where each well-received 2.5 mL of a given prey concentration plus two adult copepods for the grazer treatment, but no copepods added for the no-grazer control. Copepods were previously starved (24 h) to avoid potential effects due to variable gut fullness. Prey were diluted to the desired concentration using WC medium. Each treatment was replicated four times (except for a single treatment in the functional response experiment, the 0.125 mg C L^−1^ *Chlamydomonas*, which was replicated three times). Prey concentrations were estimated using the regression between phytoplankton biovolume and carbon biomass [82,83] using the measurements (via microscopy) of 50 cells or filaments for each prey type. Following the experiments, all copepods were alive and active.

Prey selection was calculated by selectivity coefficient (αi) using the normalized Ivlev’s ratio, which compares the relative ingestion of a given prey to its relative availability [70]. The Ivlev’s ratio for a given prey *i* (I*_i_*) is calculated by the formula I*_i_* = r*_i_* × n*_i_*^−1^, which is the clearance rate on prey *i* divided by the sum of clearance rates on all prey (r*_i_*) divided by the proportion of prey *i* among total prey (n*_i_*). We calculated the selectivity coefficient for *Raphidiopsis* (α_R_) by normalizing the Ivlev’s coefficient for this prey using the formula α_R_ = I_C_ (I_C_ + I_R_)^−1^ where I_C_ and I_R_ are the Ivlev’s ratio for the edible prey *Chlamydomonas* and *Raphidiopsis*, respectively. The selectivity coefficient is calculated for a given species and varies between zero and one, with 0.5 being no selection for a diet with two prey items [70]. Hence, values higher than 0.5 indicate positive selection (i.e., ingestion > availability) while values below 0.5 indicate avoidance (ingestion < availability).

### 5.7. Statistical Analysis

The effect of prey traits and the proportion of cyanobacteria in the diet on clearance rates or selectivity coefficients were compared using Generalized Linear Models with a gaussian family function using R software (2015, R Foundation for Statistical Computing, Vienna, Austria, version 3.1.3) [84]. Clearance rates or selectivity were the response variables while prey type (*Chlamydomonas* vs. *Raphidiopsis*), cyanobacterial defensive traits (toxin, size), and the dominance of the cyanobacterium were the independent variables.

## Figures and Tables

**Table 1:** Relative effect of prey type, i.e., *Chlamydomonas* or each of the *Raphidiopsis* strains with contrasting defensive traits (filament size: short or long; saxitoxin production: STX+ or STX−), and prey concentration (conc) on clearance rates in the functional response experiment. Prey effects are shown A) across all diets and relative to the edible prey *Chlamydomonas*; and B) across cyanobacterial diets only and relative to the long STX- strain of *Raphidiopsis*.

**A. All Prey**	**Slope**	**SE**	***t* Value**	***p***
Intercept	0.399	0.017	23.157	<0.001
Long STX-	−0.363	0.020	−17.932	<0.001
Long STX+	−0.368	0.020	−18.185	<0.001
Short STX-	−0.304	0.020	−15.055	<0.001
Short STX+	−0.359	0.020	−17.739	<0.001
Conc	−0.006	0.018	−0.354	0.725
**B. Only Cyanobacteria**	**Slope**	**SE**	***t* Value**	***p***
Intercept	0.037	0.006	6.078	<0.001
Long STX+	−0.005	0.007	−0.713	0.478
Short STX-	0.058	−0.007	8.118	<0.001
Short STX+	0.003	0.007	0.544	0.588
Conc	−0.008	0.007	−1.143	0.258

**Table 2:** The effects of *Raphidiopsis* dominance (%R) and defensive traits (filament size or toxin production) on the clearance rate of A) *Raphidiopsis* (CR_R_), B) the edible prey *Chlamydomonas* (CR_C_), C) sum of both prey, and D) sum of both prey excluding the short STX- treatment in the mixed prey experiment. Defensive trait effects show how the treatments with the long or STX+ filaments of *Raphidiopsis* changed clearance rates compared to treatments with the short or STX- filaments of *Raphidiopsis*, respectively.

**A.**	**Slope**	**SE**	***t* Value**	***p***
Intercept	0.010	0.011	0.907	0.369
%R	<0.001	<0.001	2.470	0.017
Size	−0.038	0.008	−4.802	<0.001
Toxin	−0.034	0.008	−4.357	<0.001
**B.**	**Slope**	**SE**	***t* Value**	***p***
Intercept	0.495	0.0359	13.780	<0.001
%R	−0.003	<0.001	−5.295	<0.001
Size	<0.001	0.024	0.003	0.998
Toxin	−0.0331	0.024	−1.384	0.173
**C.**	**Slope**	**SE**	***t* Value**	***p***
Intercept	0.506	0.039	12.905	<0.001
%R	−0.002	0.001	−4.097	<0.001
Size	−0.038	0.026	−1.466	0.149
Toxin	−0.067	0.026	−2.600	0.013
**D.**	**Slope**	**SE**	***t* Value**	***p***
Intercept	0.486	0.038	12.524	<0.001
%R	−0.003	<0.001	−5.075	<0.001
Size	0.028	0.031	0.914	0.368
Toxin	−0.004	0.031	−0.134	0.894

**Table 3:** The effects of *Raphidiopsis* dominance (% R) and defensive traits (size or toxin production) on the selectivity coefficient of *Raphidiopsis* (αR) in A) all treatments and B) without considering the short STX- treatment. Defensive trait effects show how the treatments with the long or STX+ filaments of *Raphidiopsis* changed αR compared to treatments with the short or STX- filaments of *Raphidiopsis*, respectively.

**A.**	**Slope**	**SE**	**t Value**	***p***
Intercept	0.187	0.028	6.576	<0.001
%R	−0.001	0.000	−3.798	<0.001
Size	−0.074	0.018	−3.943	<0.001
Toxin	−0.065	0.018	−3.446	0.001
**B.**	**Slope**	**SE**	**t Value**	***p***
Intercept	0.151	0.029	5.074	<0.001
%R	−0.001	0.000	−3.298	0.002
Size	−0.019	0.024	−0.807	0.425
Toxin	−0.010	0.024	−0.421	0.676

## Supplementary Materials

The following are available online at https://www.mdpi.com/2072-6651/12/7/465/s1, Figure S1: Design of the selective grazing experiment showing a single replicate of each treatment with a specific combination of contrasting cyanobacterial defensive traits crossed with a specific cyanobacterial dietary proportion (mixed with the edible alga *Chlamydomonas* in a total prey concentration of 0.5 mgC L^−1^) in paired experimental units with (indicated by black copepod icon) or without copepods (i.e., no-grazer control).
