# Supplementary material for: Copepod Prey Selection and Grazing Efficiency Mediated by Chemical and Morphological Defensive Traits of Cyanobacteria
Source: Toxins (Basel). 2020 Jul 21;12(7):465. doi: 10.3390/toxins12070465 (PMC7404970; doi:10.3390/toxins12070465)
Supplement: Supplementary file 1 [file toxins-12-00465-s001.pdf]

Supplementary Material: Copepod Prey Selection and Grazing Efficiency Mediated by Chemical and Morphological Defensive Traits of Cyanobacteria

Luciana M. Rangel, Lúcia H. S. Silva, Elisabeth J. Faassen, Miquel Lürling and Kemal Ali Ger

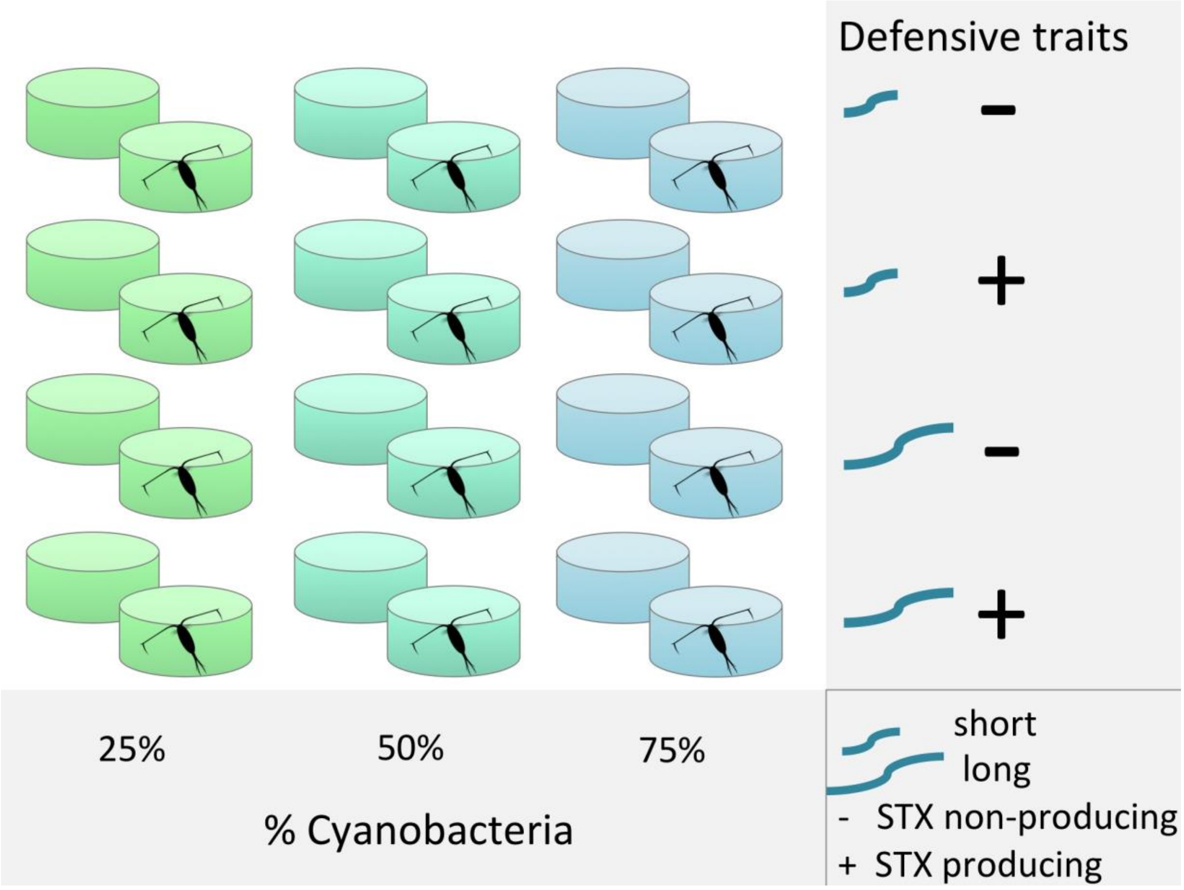

Figure S1. Design of the selective grazing experiment showing a single replicate of each treatment with a specific combination of contrasting cyanobacterial defensive traits crossed with a specific cyanobacterial dietary proportion (mixed with the edible alga *Chlamydomonas* in a total prey concentration of 0.5 mgC L^{-1}) in paired experimental units with (indicated by black copepod icon) or without copepods (i.e., no-grazer control). The cyanobacterial defensive traits were morphological (short vs. long filament size) and chemical (saxitoxin producing and non-producing). Each treatment had four replicates. See methods for details.
